# A Portable and Disposable Electrochemical Sensor Utilizing Laser-Scribed Graphene for Rapid SARS-CoV-2 Detection

**DOI:** 10.3390/bios14010010

**Published:** 2023-12-23

**Authors:** Runzhong Wang, Bicheng Zhu, Paul Young, Yu Luo, John Taylor, Alan J. Cameron, Christopher J. Squire, Jadranka Travas-Sejdic

**Affiliations:** 1 Centre for Innovative Materials and Health, School of Chemical Sciences, The University of Auckland, Private Bag 92019, Auckland 1142, New Zealand; rwan316@aucklanduni.ac.nz (R.W.); bicheng.zhu@auckland.ac.nz (B.Z.); 2 School of Chemical Sciences, The University of Auckland, Private Bag 92019, Auckland 1142, New Zealand; alan.cameron@auckland.ac.nz; 3 MacDiarmid Institute for Advanced Materials and Nanotechnology, Victoria University of Wellington, Wellington 6012, New Zealand; 4 School of Biological Sciences, The University of Auckland, Private Bag 92019, Auckland 1142, New Zealand; p.young@auckland.ac.nz (P.Y.); ja.taylor@auckland.ac.nz (J.T.); c.squire@auckland.ac.nz (C.J.S.); 5 Maurice Wilkins Centre for Molecular Biodiscovery, The University of Auckland, Auckland 1142, New Zealand; 6 Micro- and Nano-Technology Research Center, State Key Laboratory for Manufacturing Systems Engineering, Xi’an Jiaotong University, Xi’an 710049, China; yuluo825@xjtu.edu.cn

**Keywords:** laser-scribed graphene, biosensor, nanobody, electrochemistry, COVID-19

## Abstract

The COVID-19 pandemic caused by the virus SARS-CoV-2 was the greatest global threat to human health in the last three years. The most widely used methodologies for the diagnosis of COVID-19 are quantitative reverse transcription polymerase chain reaction (RT-qPCR) and rapid antigen tests (RATs). PCR is time-consuming and requires specialized instrumentation operated by skilled personnel. In contrast, RATs can be used in-home or at point-of-care but are less sensitive, leading to a higher rate of false negative results. In this work, we describe the development of a disposable, electrochemical, and laser-scribed graphene-based biosensor strips for COVID-19 detection that exploits a split-ester bond ligase system (termed ‘EsterLigase’) for immobilization of a virus-specific nanobody to maintain the out-of-plane orientation of the probe to ensure the efficacy of the probe-target recognition process. An anti-spike VHH E nanobody, genetically fused with the EsterLigase domain, was used as the specific probe for the spike receptor-binding domain (SP-RBD) protein as the target. The recognition between the two was measured by the change in the charge transfer resistance determined by fitting the electrochemical impedance spectroscopy (EIS) spectra. The developed LSG-based biosensor achieved a linear detection range for the SP-RBD from 150 pM to 15 nM with a sensitivity of 0.0866 [log(M)]^−1^ and a limit of detection (LOD) of 7.68 pM.

## 1. Introduction

In December 2019, a respiratory infectious disease later called COVID-19 was reported in Wuhan, China [1,2]. Fast and accurate diagnostic methods have proven indispensable in tracking the spread of the virus during the pandemic and in the isolation of infected cases to mitigate the spread of the virus and its impact on societies. For disease control and prevention, disposable and single-use point-of-care methodologies are crucial for early diagnosis. Serological antibody tests using enzyme-linked immunoassay (ELISA) are another option for COVID-19 diagnosis. These are commonly used as supplementary tests for negative RT-PCR cases [3]. ELISA employs the recombinant spike protein and nucleocapsid protein as the probes to detect antibodies of COVID-19 IgG/IgM. Zhang et al. used anti-human IgG-HRP (horseradish peroxidase) conjugated monoclonal antibody and anti-human IgM for IgG/IgM test. They noted an increasing IgM/IgG positive rate (50% to 100%) after day 5 and it was almost undetectable on day 0. They also found that an anal swab was more reliable at an early stage of infection compared to an oral swab [4]. The presence of IgM in the sample indicates a recent infection by COVID-19. The paper-based ELISA is a fast, cheap, and portable diagnostic method that can be used for point-of-care tests [5]. However, this technology does not provide a quantitative analysis. The sensitivity of ELISA for the combined IgM/IgG test is 87.3%, which is relatively low compared to RT-PCR tests [6]. Another lateral flow technology for COVID-19 detection is the rapid antigen test (RAT) methodology, which is currently widely used for rapid COVID-19 tests at home [7]. The mechanism of RAT is that a specimen analyte, containing SARS-CoV-2 nucleocapsid (N) antigen suspended in an assay buffer, is deposited in the sample well. The specimen sample diffuses to the conjugate pad where nucleocapsid (N) antigen binds with the AuNPs-modified anti-nucleocapsid protein antibody. This process results in a color change in the test line due to the presence of AuNPs. The RAT test is quick and simple; however, it does not provide a quantitative result and is of limited sensitivity (CT value of 25 for the detection of COVID-19 [7]).

As ELISA- and RAT-based disposable strip detection methods all have their inherent limitations, alternative methodologies continue to be developed for portable, rapid, accurate, and sensitive detection of COVID-19 and similar infectious diseases. Biosensors based on field-effect transistors (FET) are an attractive option in clinical diagnosis, point-of-care testing, and on-site detection [8]. Seo et al. developed a field-effect transistor (FET) biosensor for the rapid detection of COVID-19 virus where SARS-CoV-2 spike antibody (IgG) was anchored on the graphene channel as a probe for the detection of the whole virus [9]. The LoD of this FET device was reported as 1 fg/mL for the spike protein and 2.42 × 10^2^ copies/mL for the whole virus in clinical samples. Electrochemical biosensors are also attractive candidates for point-of-care COVID-19 tests since they are cost-efficient and easy to operate. Yakoh et al. reported an electrichemical paper-based analytical device (ePAD) for diagnosing COVID-19 [10]. The working electrode was prepared from graphene oxide followed by the attachment of SARS-CoV-2 antibodies (both IgG and IgM) on the graphene oxide through EDC/NHS chemistry. The SARS-CoV-2 spike protein containing the receptor-binding domain (SP-RBD) was captured by SARS-CoV-2 antibodies on the ePAD and the signal was measured by means of square-wave voltammetry (SWV). This sensor provided a linear detection range toward the spike protein between 1–1000 ng/mL with a LOD of 0.11 ng/mL.

Laser scribed graphene (LSG) is made by direct laser scribing on a polymer substrate, such as polyimide (PI), leading to C–O, C–N and C=O bonds breaking and re-forming, with a release of CO_2_/NO_2_/H_2_O gases. The remaining aromatic carbons form graphene [11]. Compared to the graphene FET sensor [9] and other disposable paper-based electrochemical sensors [10,12,13], the fabrication of laser-scribed graphene electrodes is simple and time- and cost-efficient and the electrodes can be easily patterned; thus, the process is amenable to mass production. The average cost of an LSG electrode was ca. NZD 0.23. By comparing the cost of an FET device, including the cost of the silicon wafer, the cost of a mask for photolithography, and usage charges for a clean room for photolithography, LSGEs are much cheaper and more cost-effective. LSG electrodes have been employed in a number of applications, including in supercapacitors [14], hydrogen evolution [15], and biosensors [16]. Beduk et al. reported a gold-coated LSG electrode for SARS-CoV-2 S1 spike protein detection where the LSG electrode surface was modified by electrochemically deposited AuNPs [17]. SARS-CoV-2 spike protein antibody was anchored onto the AuNPs. The biorecognition event was evaluated by differential pulse voltammetry (DPV) measurements in potassium ferri/ferrocyanide solution where the bulky spike protein bound to the electrode surface hindered the electron transfer process leading to a decrease in DPV current peak. The dynamic range was found to be in the 10–75 ng/mL range, with LoD of 2.9 ng/mL.

Nanobodies are recombinant proteins that contain the variable domain of antibodies comprising a single heavy chain only, typically produced by camels, lammas, and other members of the *Cammelidae* family [18]. These single-chain antibody fragments are advantageous as they are highly stable with a strong affinity for their cognate antigen. To the best of our knowledge, the only known biosensor using nanobody receptor for SARS-CoV-2 detection is an organic electrochemical transistor (OECT) reported by Guo et al. [19], which has not yet been applied to LSG-based biosensors. In their design of the nanobody-based OECT sensor, they employed a nanobody-SpyCatcher fusion protein as a probe for COVID-19 and MERS antigens detection. In the OECT-gate functionalization process and the 7-methoxycoumarin-4-acetic acid (MCA)-modified SpyTag peptide was immobilized on the 1,6-hexanedithiol self-assembly monolayer through a flexible linker on top of a gold gate electrode. The SpyCatcher domain could specifically bind to SpyTag peptide through forming an isopeptide bond. The SpyCatcher domain was then fused with a spike-specific nanobody through a 12-nm-long flexible linker. This SpyTag/SpyCatcher system helped to control the orientation of the nanobody during immobilization and improved the sensor performance. Their devices provided fast readout, within 10 min of incubation with clinic nasopharyngeal swabs and saliva samples. The limit of detection for SARS-CoV-2 S1 protein (in universal transport medium) was 1.453 pg/mL.

In this study, a disposable single-use electrochemical biosensor for the detection of SARS-CoV-2 spike protein was developed. The disposable laser-scribed graphene electrodes were produced and modified by immobilizing SARS-CoV-2 spike protein utilizing a split-ester bond ligase system [20]. Firstly, an ester bond peptide tag (E-Tag) was modified with a C-terminal pyrene and immobilized on the graphene electrode. A spike-specific VHH E nanobody [21] genetically fused with a C-terminal ester bond ligase (EsterLigase) was subsequently captured and covalently ligated to the electrode via a spontaneous autocatalytic ester bond formed between the E-Tag and EsterLigase. The presence of the SARS-CoV-2 spike protein containing the receptor-binding domain (SP-RBD) was then measured by electrochemical impedance spectroscopy (EIS) in a buffer solution containing a ferri/ferrocyanide (Fe[(CN)_6_]^3−/4−^) redox couple. A linear detection range from 150 pM to 15 nM, a LoD of 7.68 pM, and excellent selectivity towards other potential viral proteins were demonstrated.

## 2. Materials and Methods

### 2.1. Materials and Chemicals

Potassium ferricyanide (III), potassium ferrocyanide (II) trihydrate, and phosphate buffered saline (PBS) tablets were purchased from Sigma-Aldrich (Burlington, MA, USA). 4-(Pyren-1yl) butanoic acid (PBA) and 4-(2-hydroxyethyl)-1-piperazineethanesulfonic acid (HEPES) were purchased from AK Scientific Inc (Union city, CA, USA). SP-RBD, Influenza H3N2, and H1N1 were purchased from Thermo Fisher Scientific (Waltham, MA, USA). The chemicals and materials used for EsterLigase-VHH E production pyrene-E-Tag synthesis are provided in Supporting Information S1.

### 2.2. EsterLigase-VHH E Production

The split-ester bond ligation system was identified and developed using previously described protocols [22]. Briefly, an intramolecular ester bond-containing domain was identified in a cell surface adhesin from *Gemella bergeriae* (ATCC 700627). The domain (residues 732-882, Uniprot U2Q1B9) was split at residue Asp 862 to produce an N-terminal EsterLigase and a C-terminal E-Tag that, when mixed covalently, ligate together through a spontaneous ‘intramolecular’ ester bond as the domain reassembles.

The VHH E nanobody [21] EsterLigase fusion protein (Figure S1A) was synthesized as a gBlock (GenScript, Piscataway, NJ, USA) cloned into the expression vector pProExHta with In-Fusion^®^ cloning and transformed into chemically competent Stellar™ *E. coli* cells (Clontech, Mountain View, CA, USA). A single colony was cultured overnight in 10 mL of 2×YT medium at 30 °C and transferred to 2 L baffled culture flasks containing 1 L of 2×YT medium supplemented with 0.1 mg/mL ampicillin. Cultures were grown at 30° with shaking to an OD600 of ~0.8 before induction with 0.3 mM isopropyl-β-D-1-thiogalctopyranoside (IPTG). The culture was transferred to 18 °C and incubated for 16 h before harvesting by centrifugation. The cell pellet was re-suspended in 20 mL lysis buffer (50 mM Tris.Cl pH 8.0, 500 mM NaCl, 10 mM imidazole, and 0.5 mM TCEP), supplemented with complete protease inhibitor cocktail mini tablets (EDTA free; Roche), and transferred to 50 mL falcon tubes and stored at −20 °C.

Cell pellets were lysed using a cell disruptor (Constant Cell Disruption Systems) at 124 kPa and the lysate was clarified by centrifugation at 30,000× *g* for 20 min at 4 °C. The soluble protein was applied to a 5 mL IMAC column (HiTrap) and bound VHH E nanobody EsterLigase protein was washed with wash buffer (50 mM Tris.Cl pH 8.0, 300 mM NaCl, 20 mM imidazole) and eluted in a linear gradient with elution buffer (50 mM Tris.Cl pH 8.0, 300 mM NaCl, 500 mM imidazole). The N-terminal vector-derived polyhistidine tag was cleaved from VHH E nanobody EsterLigase protein with a 1:100 ratio of recombinant Tobacco Etch Virus (rTEV) protease to recombinant protein and concurrently dialyzed against 1 L buffer (10 mM Tris-Cl [pH 8.0], 100 mM NaCl, 1 mM β-mercaptoethanol) at 4 °C for 16 h. Cleaved VHH E nanobody EsterLigase protein was separated from the rTEV-His6 protease by subtractive IMAC. The unbound protein containing cleaved VHH E nanobody EsterLigase protein was collected and concentrated using a 30-kDa-molecular-weight-cutoff (MWCO) protein concentrator (VivaScience) and purified by size exclusion chromatography on a Superdex S200 10/300 column (GE Healthcare). Purified VHH E nanobody EsterLigase eluted as a single peak and was judged to be approximately >99% pure, as inferred by SDS-PAGE analysis.

### 2.3. Synthesis of Pyrene-E-Tag

The E-Tag peptide (Figure S1B) was prepared by Fmoc solid-phase peptide synthesis (Fmoc-SPPS) [23], with coupling reactions performed under microwave irradiation for 5 min at 70 °C using a Biotage Biotage^®^ Initiator+ Alstra™ (Uppsala, Sweden). All other chemical manipulations were undertaken at room temperature. Briefly, the peptide was prepared as the corresponding C-terminal amide derivative using Fmoc-Rink amide linker with TentaGel S NH_2_ resin (0.23 mmol/g, Rapp Polymere, Tubingen, Germany). The E-Tag complement peptide sequence was modified to contain a GSGSGAKG spacer sequence at its *C*-terminus. The lysine residue of this sequence was incorporated with orthogonal side chain protection as Fmoc-Lys(Dde)-OH. Upon completion of the linear peptide sequence, the N-terminal Fmoc group was exchanged for Boc protection on-resin by treatment with Boc_2_O in DMF [24], followed by removal of the Dde protecting group from the N^ε^ amino group of the most C-terminal lysine with a 2% hydrazine solution in DMF [25]. The free amino group was coupled 4-(pyren-1-yl)butanoic acid (5 equiv., AK Scientific, Union City, CA, USA) with HATU (4.8 equiv.) for 30 min at rt. Upon resin cleavage, the crude peptide was purified by RP-HPLC to afford the title compound in 25.6% overall yield (based on resin loading) in 95.5% purity (Figure S2). The identity of the pyrene-E-Tag was confirmed by mass spectrometry (Figure S3).

### 2.4. Fabrication of LSG Electrodes

The laser-scribed graphene (LSG) electrodes on the polyimide (PI) sheet were used as sensing strips for the impedimetric detection of SP-RBD. The LSG electrodes were fabricated using a universal laser system (laser cutter VLS3.50, Product Development Inc., Knoxville, TN, USA) equipped with a CO_2_ laser. The fabrication method and optimized laser parameters were previously reported by Zhu et al. [16]. In short, the wavelength of the laser was set to 10.6 μm, the optimized lens substrate distance was 5.1 cm, the laser speed was 0.45 cm/s, the power was 2.7 W, and the resolution (pulses per centimeter) was 393.7. The laser scribing process was conducted in a vacuum chamber.

Each LSG sensing strip contained an LSG working electrode (WE), an LSG counter electrode (CE), and an LSG electrode with Ag paste painted and dried in an oven at 60 °C for 30 min as the reference electrode (RE). The diameter of the working electrode was 2 mm. A dielectric layer was applied on the electrode tracks to prevent the spreading of the solution.

The fabricated LSG electrodes were characterized by scanning electron microscopy (SEM) using a Philip ML30S FEG scanning electron microscope (Amsterdam, the Netherlands). The LSG electrodes were sputter-coated with 20 nm gold before imaging.

The Raman spectrum (Horiba Japan) of the LSG is presented in Figure S4. The excitation wavelength was 523 nm. The characteristic D (1344 cm^−1^), G (1576 cm^−1^) and 2D (2690 cm^−1^) peaks of graphene were observed.

### 2.5. Functionalization of LSG Electrodes

A stock solution (500 μM) of 4-(pyren-1yl) butanoic acid (PBA) was made by dissolving 4.3 mg of PBA in 30 mL of ethanol with sonication. This PBA solution was diluted 100 times with ethanol and then 10 times with PBS to obtain 500 nM of PBA in a mixture of PBS and ethanol (9:1 vol:vol). A 500 nM solution of the pyrene-E-Tag linker was prepared in a PBS buffer. To maintain the space between pyrene-E-Tag linkers and EsterLigase-nanobody, 10 μL of 500 nM PBA solution, 10 μL of 500 nM pyrene-E-Tag linker PBS solution, and 80 μL PBS buffer were mixed, providing a final solution containing 50 nM pyrene-E-Tag linker and 50 nM PBA. The LSG working electrode was first incubated with 20 μL of 50 nM pyrene-E-Tag linker/PBA (1:1 mol/mol, 50 nM each) mixture for 3 h at room temperature and then washed with PBS buffer to remove the unbound pyrene-E-Tag linker and PBA (referred to here as “LSG/PBA-pyrene-E-Tag linker”). The LSG/PBA-pyrene-E-Tag linker was incubated with 20 μL of 2.5 μM EsterLigase-nanobody in 50 mM HEPES solution containing 20 vol% glycerol overnight at 4 °C in the fridge; the resulting modified electrode is referred to here as “LSG/PBA-pyrene-E-Tag linker/EsterLigase-nanobody”. Glycerol was used as it was shown that it can facilitate the ligation reaction by stabilizing the nanobody structure and preventing aggregation [26]. The electrode was washed with PBS buffer 3 times to remove the unreacted nanobody before the detection of SP-RBD.

### 2.6. SP-RBD Detection by Electrochemical Impedance Spectroscopy (EIS)

The LSG/PBA-pyrene-E-Tag linker/EsterLigase-nanobody was incubated with 20 μL of SP-RBD of various concentrations in PBS solution for 30 min at room temperature. After incubation with SP-RBD, LSG/PBA-pyrene-E-Tag linker/EsterLigase-nanobody was then washed with PBS buffer thoroughly. The EIS was carried out using PalmSens 4. The EIS measurements were performed in PBS buffer, containing 5 mM Fe[(CN)_6_]^3−/4−^ (1:1 mol:mol) as a redox couple, with a bias potential of 0.14 V (vs. Ag reference electrode) and an amplitude of 10 mV in a frequency range from 100 kHz to 0.1 Hz.

### 2.7. Fabrication of Low-Dimensional Graphene and AFM Characterization of Surface Modification

To construct a flat 2D graphene surface for AFM measurements, a graphene oxide (GO) film was prepared on 200 nm SiO_2_/Si substrate using an electrospraying technique. The solution consisted of 5 mg/mL graphene oxide in ethanol/propylene carbonate with a ratio of 4:3:3 (GO:ethanol:propylene carbonate). The flow rate, potential, and height for the electrospraying of GO wet film were 90 μL/h, 3.3 kV, and 15 mm, respectively. The GO film was then treated with vacuum-flash assisted evaporation for 10 min and heated up to 120 °C for 30 min to remove all solution on the surface. After the preparation of GO film, a vapor phase reduction was carried out for 8 h at 100 °C, where a mixture solution of HI/AcOH (1:1) was used as a reducing agent. The rGO film was annealed at 100 °C for 2 h to stabilize the binding between the rGO film and SiO_2_/Si substrate.

The 2D graphene surface was then modified by first treating the surface with 20 μL of 2.5 nM pyrene-E-Tag linker in PBS solution for 3 h at room temperature. After washing with PBS buffer, the graphene surface was incubated with 20 μL of 5 μM EsterLigase-nanobody in HEPES solution containing 20 vol% glycerol and stored overnight at 4 °C in the fridge. In total, 20 μL of 1.5 nM SP-RBD was added to the surface followed by 30 min of incubation before AFM measurements were taken. The AFM images were taken on separate graphene chips for each step of the modification process.

The AFM imaging of the graphene surface was conducted on a Cypher ES AFM (Asylum Research, Santa Barbara, CA, USA) in tapping mode using a silicon tip. The resonance frequency of the tip was 150 kHz. The samples were transferred to a 15 mm-diameter AFM specimen disc using double-sided tape.

### 2.8. FT-IR Characterization of LSG Electrode Surface Modification with Pyrene-E-Tag Linker 

The attenuated total reflectance (ATR)-FTIR characterization was carried out by an FTIR Bruker Vertex 70 spectrometer (Billerica, MA, USA). The bare LSG electrode was modified with 20 μL of 250 nM pyrene-E-Tag linker in PBS solution for 3 h and then washed with PBS buffer for 3 times before FT-IR measurement.

## 3. Results and Discussion

### 3.1. Design of an LSG-Based Electrochemical Biosensor for the Detection of SARS-CoV-2 Spike Protein RBD

**Figure 1:**
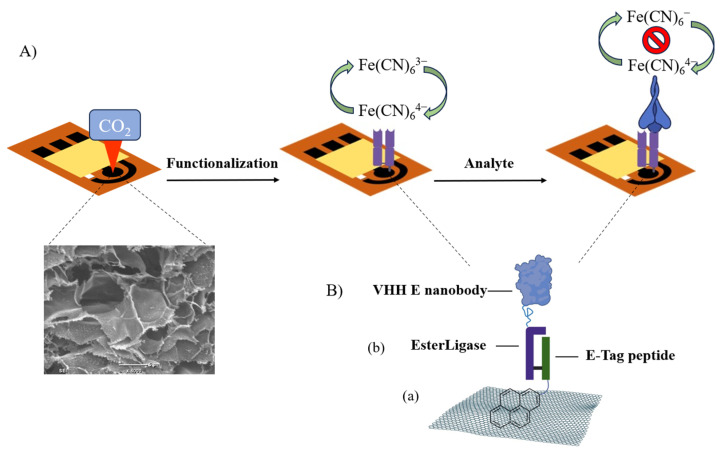
(**A**) Scheme of the construction of LSG-based SARS-CoV-2 spike RBD protein biosensor for the detection of SP-RBD. (**B**) Surface modification of the LSG electrode including (**a**) the immobilization of pyrene-modified E-Tag peptide to the LSG surface via Π–Π stacking. (**b**) The conjugation between EsterLigase and E-Tag through the formation of an ester bond. The mechanism of conjugation employed was as described by Young et al. [20].

A schematic of the LSG-based biosensor design for the detection of SP-RBD is presented in Figure 1A. The pyrene-E-Tag linker and PBA spacer were assembled onto the laser-scribed graphene (LSG) surface via Π–Π stacking [27]. The pyrene-E-Tag linker can be specifically recognized by EsterLigase, which was fused with the anti-spike VHH E nanobody. EsterLigase can spontaneously form an ester bond with E-Tag peptide, formed between Thr of the EsterLigase and Gln of E-tag side chains [20], thereby modifying the working electrode with the nanobody and preserving the vertical orientation of the nanobody. The anti-spike VHH E nanobody, one of the variable domains of heavy-chain-only antibodies (VHHs) derived from llama [21], acted as the bio-recognition element for the detection of SP-RBD (Figure 1B). The biorecognition between the target and the nanobody is similar to antigen and antibody recognition; however, the nanobody is much smaller than an antibody [21]. The binding between the pyrene-E-Tag linker and the nanobody was validated by sodium dodecyl-sulfate polyacrylamide gel electrophoresis (SDS-PAGE) (Figure S5). The result indicated the formation of a larger species after mixing the anti-spike VHH E nanobody with the pyrene-E-Tag linker, confirming the successful binding of EsterLigase-nanobody to the pyrene-E-Tag linker.

**Figure 2:**
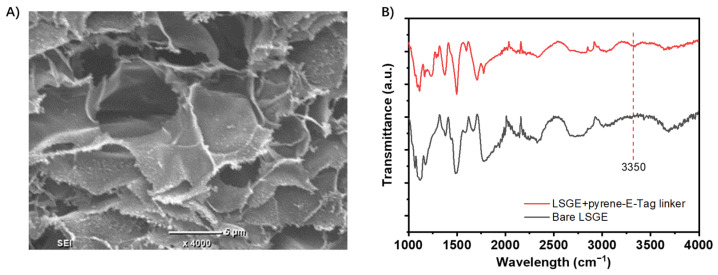
(**A**) SEM image for bare LSG electrode. (**B**) FTIR spectra of bare LSG electrode and LSG electrode functionalized with a pyrene-E-Tag linker.

The morphology of the bare LSG electrodes was characterized by SEM imaging (Figure 2A). The highly porous structure of the LSG electrode can be observed, which indicates a high surface area for surface functionalization [16]. FTIR spectrum of the LSG electrode before and after the modification with the pyrene-E-Tag linker (Figure 2B) confirms the successful attachment of the pyrene-E-Tag linker on the LSG surface. A new peak around 3350 cm^−1^, corresponding to the N-H stretch from the secondary amine within pyrene-E-Tag linker, was observed after the modification.

**Figure 3:**
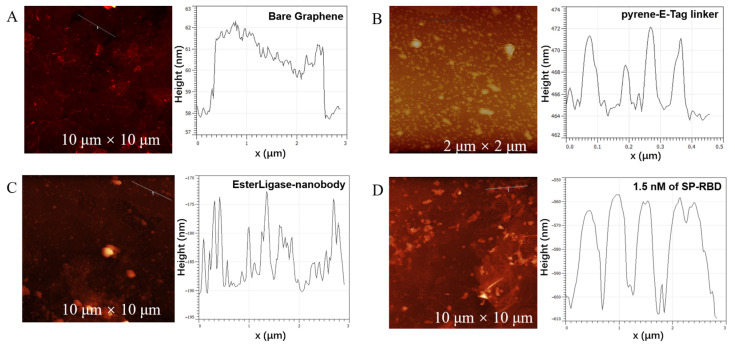
AFM images for each step of the graphene surface modification. (**A**) Bare graphene surface. Graphene surface modified with (**B**) pyrene-E-Tag linker, (**C**) pyrene-E-Tag linker/EsterLigase-nanobody, and (**D**) after incubation with 1.5 nM of SP-RBD for 30 min.

AFM was employed to characterize the functionalization of the working electrode. Since the porous LSG was not ideal for AFM studies, a low-dimensional graphene surface was fabricated for AFM measurements. The bare graphene layer had a thickness of 3 ± 0.5 nm (Figure 3A), while the pyrene-E-Tag linker functionalized graphene had a thickness of ~5 nm (Figure 3B). After the conjugation of EsterLigase-nanobody, the thickness increased to 10–15 nm, where the thickness of EsterLigase-nanobody was ca. 5–10 nm, calculated by subtracting the thickness of pyrene-E-Tag linker (Figure 3C). The thickness of SP-RBD was estimated as 25–30 nm in Figure 3D. The increment in thickness from 3 nm for bare graphene to ~40 nm after all steps of modification indicates a successful functionalization of the graphene surface.

### 3.2. Feasibility of SARS-CoV-2 Sensing Strip to Detect SP-RBD Protein

**Figure 4:**
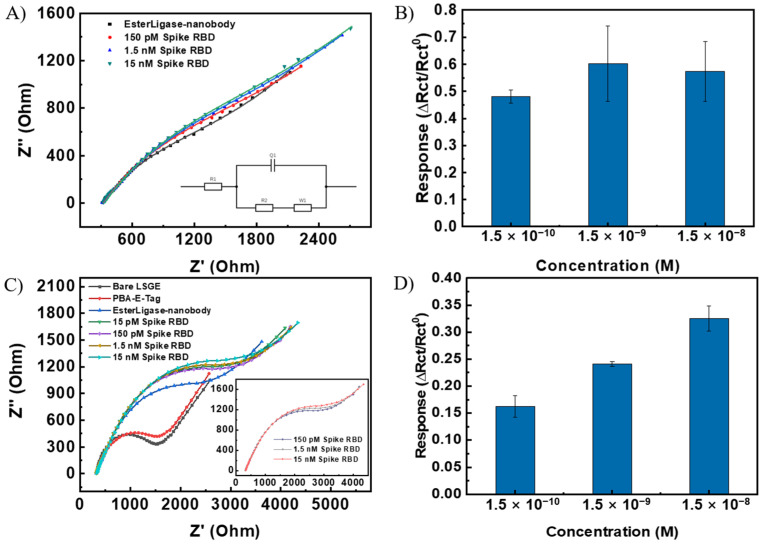
(**A**) Nyquist plot of LSG/pyrene-E-Tag linker/EsterLigase-nanobody electrode after the detection of SP-RBD in concentrations from 150 pM to 15 nM. (**B**) Normalized response, ∆*R*_ct_/*R*_ct_^0^, of LSG/pyrene-E-Tag linker/EsterLigase-nanobody for the detection of SP-RBD from 150 pM to 15 nM. (**C**) Nyquist plot of LSG/PBA-pyrene-E-Tag linker/EsterLigase-nanobody electrode after the detection of SP-RBD detection from 15 pM to 15 nM (150 pM to 15 nM is shown as inset). (**D**) ∆*R*_ct_/*R*_ct_^0^ of LSG/PBA-pyrene-E-Tag linker/EsterLigase-nanobody after the detection of SP-RBD protein from 150 pM to 15 nM. The sensitivity has been improved after using PBA for modification. (Error bars were obtained from the standard deviations of 3 independent experiments).

After confirming the successful functionalization of the EsterLigase-nanobody onto the LSG electrode, the binding between the anti-spike VHH E nanobody and SP-RBD was investigated by EIS, as shown in Figure 4. The Randle’s equivalent circuit model was used to fit the spectra (Figure 4A inset). It consisted of solution resistance (*R*_s_), charge transfer resistance (*R*_ct_), constant phase element (*Q*_1_), and Warburg resistance (*W*_1_). The normalized change in charge transfer resistance (Δ*R*_ct_/*R*_ct_^0^) before and after the detection of SP-RBD was used as the sensor response, where *R*_ct_^0^ represents the charge transfer resistance of LSG/PBA-pyrene-E-Tag linker/EsterLigase-nanobody. After the modification of LSG, the LSG/PBA-pyrene-E-Tag linker/EsterLigase-nanobody was washed with PBS three times to remove the un-bound EsterLigase-nanobody (Figure S6), with no change in the EIS readout after each washing. After the detection of SP-RBD, an increase in *R*_ct_ was observed due to the blockage of Fe[(CN)_6_]^3−/4−^ to access the LSG/PBA-E-Tag /EsterLigase-nanobody.

The necessity of the use of 1-pyrene butyric acid (PBA) to construct a successful sensing platform was investigated. For LSG/pyrene-E-Tag linker/EsterLigase-nanobody sensors where PBA was not used, the semicircle of the Nyquist plot (representing the charge transfer process on the LSG surface [28]) is not obvious (Figure 4A), with the ∆*R*_ct_/*R*_ct_^0^ signal presented in Figure 4B. The signal only slightly increased from 0.48 for 150 pM SP-RBD to 0.65 for 15 nM SP-RBD. The large semicircles indicate slow charge transfer kinetics due to a high blockage of highly dense EsterLigase-nanobody on the LSG surface. The standard deviations (error bars) after detection of SP-RBD on the LSG/pyrene-E-Tag linker/EsterLigase-nanobody electrode was large (Figure 4B), indicating the poor repeatability of the sensor response from independent sensor strips. To improve the repeatability, PBA, which also contains a pyrene group for self-assembling onto the LSG surface through Π–Π stacking, was mixed with the pyrene-E-Tag linker (1:1 mol:mol, 50 nM each) in order to space out the EsterLigase-nanobodies on the LSG surface and to improve their orientation. When a mixture of PBA and pyrene-E-Tag linker (1:1 mol:mol, 50 nM each) was used for the LSG surface modification, the semicircle in the Nyquist plot of EIS (Figure 4C) is more obvious, which indicates a stronger and faster charge transfer reaction of Fe[(CN)_6_]^3−/4−^ on the LSG surface. The sensor responses, ∆*R*_ct_/*R*_ct_^0^, were from 0.16 to 0.31 after the detection of 150 pM to 15 nM SP-RBD (Figure 4D). The value of the sensing response (∆*R*_ct_/*R*_ct_^0^) of LSG/PBA-pyrene-E-Tag linker/EsterLigase-nanobody was smaller (Δ*R*_ct_/*R*_ct_^0^ = 0.31) after the detection of 15 nM SP-RBD compared with LSG/pyrene-E-Tag linker/EsterLigase-nanobody without PBA (Δ*R*_ct_/*R*_ct_^0^ = 0.65). We suggest these results from the smaller amount of anti-spike VHH E nanobodies on the LSG surface when the mixed pyrene-E-Tag linker and PBA monolayer were assembled on the surface. Three different molar ratios of PBA to pyrene-E-Tag linker were tested, as shown in Figure S7. The impedance spectra from the molar ratio of 1:1 (PBA: pyrene-E-Tag linker) showed a more obvious semicircle, compared to ratios of 2:1 and 1:2.

**Figure 5:**
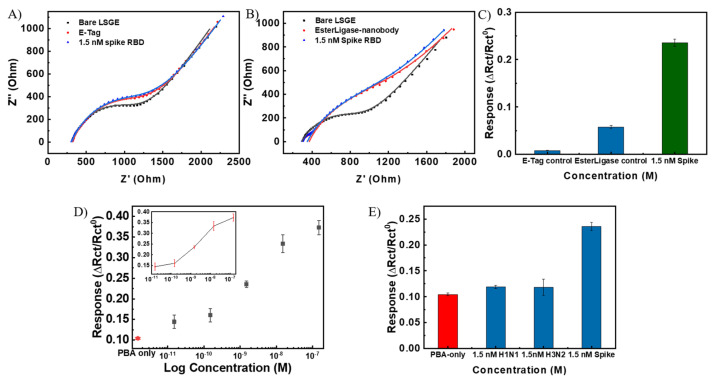
Nyquist plot of (**A**) LSG/PBA/EsterLigase-nanobody and (**B**) LSG/ LSG/PBA-pyrene-E-Tag linker after exposure to 1.5 nM SP-RBD. (**C**) Normalized responses (∆*R*_ct_/*R*_ct_^0^) from (**A**,**B**). ∆*R*_ct_/*R*_ct_^0^ to 1.5 nM SP-RBD as a positive given for comparison. (**D**) Calibration curve of the LSG/PBA-pyrene-E-Tag linker/EsterLigase-nanobody sensor (∆*R*_ct_/*R*_ct_^0^) for the detection of SARS-CoV-2 SP- RBD at different concentrations from 15 pM to 150 nM (*n* = 3) and the control LSG electrode strip with the modification with only PBA after the detection of 1.5 nM of SP-RBD. (**E**) LSG/PBA-pyrene-E-Tag linker/EsterLigase-nanobody sensor responses, ∆*R*_ct_/*R*_ct_^0^, after exposure to H1N1 and H3N2 influenza spike proteins (*n* = 3), as controls. ∆*R*_ct_/*R*_ct_^0^ to 1.5 nM SP-RBD as a positive given for comparison. (Error bars were obtained from the standard deviations of 3 independent experiments).

The control experiments were carried out by functionalizing LSG working electrode in the absence of either the pyrene-E-Tag linker or EsterLigase-nanobody, named LSG/PBA-pyrene-E-Tag linker and LSG/PBA/EsterLigase-nanobody. The LSG/PBA-pyrene-E-Tag linker and LSG/PBA/EsterLigase-nanobody electrode strips were applied for the detection of 1.5 nM SP-RBD, as shown in Figure 5A,B. The LSG/PBA-pyrene-E-Tag linker electrode strip had a ∆*R*_ct_/*R*_ct_^0^ of 0.0080 ± 0.0004 (Figure 5C), which was negligible when compared with the ∆*R*_ct_/*R*_ct_^0^ of 0.240 ± 0.008 obtained from LSG/PBA-pyrene-E-Tag linker/EsterLigase-nanobody, indicating a specificity between EsterLigase-nanobody and SP-RBD. A ∆*R*_ct_/*R*_ct_^0^ of 0.0058 ± 0.0029 was obtained from the LSG/PBA/EsterLigase-nanobody, which was much smaller than that from LSG/PBA-pyrene-E-Tag linker/EsterLigase -nanobody, again demonstrating the specific binding between pyrene-E-Tag linker and EsterLigase-nanobody. The *R*_ct_ increased after the incubation of LSG/PBA with EsterLigase-nanobody (Figure 5B), indicating some non-specific adsorption of EsterLigase-nanobody on the LSG electrode. However, the sensor response was negligible, demonstrating the pyrene-E-Tag linker’s role in facilitating the orientation of EsterLigase-nanobody for the binding of SP-RBD and the importance of the EsterLigase-nanobody orientation on the sensor’s surface. 

### 3.3. Sensing Performance of the SARS-CoV-2 Sensing Strip

The sensing responses of the sensor to five different concentrations of SP- RBD from 15 pM to 150 nM were measured on three independent sensors. A control sensor was functionalized with PBA only instead of the mixture of PBA and pyrene-E-Tag linker and exposed to 1.5 nM of SP-RBD. The obtained dose–response curve, shown in Figure 5D, indicates a linear detection range between 150 pM to 15 nM for SP-RBD, with an LoD of 7.68 pM (3 × S/N).

To investigate the specificity of the developed LSG/PBA-pyrene-E-Tag linker/EsterLigase-nanobody sensing strip toward the detection of SP-RBD, the sensor strip was exposed to H1N1 and H3N2 influenza spike proteins as interfering targets. The results (Figure 5E) suggest that there is no binding between the influenza spike protein and EsterLigase-nanobody, with ∆*R*_ct_/*R*_ct_^0^ of 0.120 ± 0.003 and 0.120 ± 0.016 (*n* = 3) after exposure to 1.5 nM of either H1N1 or H3N2 spike proteins, respectively. These values are much smaller than the response to 1.5 nM of SP-RBD (∆*R*_ct_/*R*_ct_^0^ = 0.240 ± 0.008) and similar to the change in the signal when the sensor surface is not functionalized with the probe at all.

Table 1 summarizes the sensing responses from comparable electrochemical biosensors for SARS-CoV-2 detection. Compared to the reported sensors, such as LSG/AuNS electrodes that require 1h for target incubation [17], our design can provide a faster readout after 30 min of incubation and ca. 3 min for EIS measurement, with a lower LoD of 7.68 pM, compared to 116 nM [17]. The LSG electrode strip in this work is portable and disposable, which could be applied for point-of-care detection [13,17] and be convenient to use. Our disposable sensing strips platform has the potential for portable point-of-care diagnostic applications.

## 4. Conclusions

This study describes a disposable, portable, and electrochemical LSG-based sensor based on a bio-recognition system with a pyrene-E-Tag linker and EsterLigase-nanobody for the detection of SP-RBD. The developed sensor provides a linear detection range from 150 pM to 15 nM towards the detection of SP-RBD, with an LoD of 7.68 pM and excellent selectivity towards other potential viral proteins. The developed sensor can provide a rapid readout within 30 min. This developed sensor has the potential for rapid, sensitive, selective, and point-of-care diagnosis of SARS-CoV-2.

## Figures and Tables

**Table 1:** Comparison with various electrochemical biosensors.

Technique	Analyte	Linear Range	LoD	Ref.
μPAD, EIS	IgG	10–1000 ng/mL	0.4 pg/mL	[12]
AuNPs modified Paper electrodes	N-protein	585–5.854 × 10^7^ copies/μL	6.9 copies/μL	[13]
LSG/AuNS electrods, DPV	S-protein RBD	5–500 ng/mL	2.9 ng/mL	[17]
Co-TNTs	S-protein RBD	14–1400 nM	0.7 nM	[29]
3D nanoprinting rGO, EIS	S-protein RBD	1.0 fM–1 nM	16.9 fM	[30]
LSG, EIS	S-protein RBD	15 pM–150 nM	7.68 pM	This work

## Data Availability

Data are available upon requirement.

## Supplementary Materials

The following supporting information can be downloaded at https://www.mdpi.com/article/10.3390/bios14010010/s1. Figure S1. (A) Sequence of VHH E nanobody fused with EsterLigase. The N-terminal His^6^ IMAC tag and rTEV protease recognition sequence are underlined and removed from the purified VHH E EsterLigase recombinant protein. The VHH E nanobody sequence (grey) is linked to the *Gemella bergeriae* (Gberg) EsterLigase domain (yellow) via a flexible linker (red). (B) Structure of pyrene-modified E-Tag. The E-Tag is composed of a Gberg ligation peptide with a C-terminal spacer that is conjugated to pyrene via the lysine N^ε^ amino group; Figure S2. Analytical RP-HPLC for pyrene-modified E-Tag. RP-HPLC profile (214 nm) of pyrene modified E-Tag peptide. t_R_ 27.8 min, Phenomenex Luna C18 (100Å, 5 μm, 4.6 mm × 250 mm), linear gradient 5%B to 65%B (*ca.* 1%B/min) at 1 mL/min; Figure S3. ESI-MS data for pyrene-modified E-Tag. Pyrene modified synthetic peptide ESI-MS (+ve) data. *m*/*z* calculated for [C165H253N45O47] 3617.9; mass observed deconv: 3618.6 ± 0.13. Charge states; 604.1 [M+6H]6+, 724.7 [M+5H]5+, 905.7 [M+4H]4+, 1207.2 [M+3H]3+; Figure S4. The Raman spectrum of the LSG. Excitation wavelength: 523 nm; Figure S5. SDS-PAGE analysis (12% acrylamide gel) for ester bond formation. Lane A: EsterLigase-nanobody. Lane B: E-Tag and EsterLigase-nanobody allowed for overnight ligation reaction. Two species are visible from SDS-PAGE as the nanobody is not fully reduced; Figure S6. EIS spectra of LSG/PBA-pyrene-E-Tag linker/EsterLigase-nanobody after 1, 2 and 3 times washes with PBS buffer; Figure S7. EIS spectra from the sensors with three different molar ratios of PBA to pyrene-E-Tag linker [31].
