# Supplementary material for: A Portable and Disposable Electrochemical Sensor Utilizing Laser-Scribed Graphene for Rapid SARS-CoV-2 Detection
Source: Biosensors (Basel). 2023 Dec 23;14(1):10. doi: 10.3390/bios14010010 (PMC10813335; doi:10.3390/bios14010010)
Supplement: Supplementary file 1 [file biosensors-14-00010-s001.zip › biosensors-2765528-supplementary.pdf]

Supplementary Information

A Portable and Disposable Electrochemical Sensor Utilizing Laser-Scribed Graphene for Rapid SARS-CoV-2 Detection

Runzhong Wang [1,†], Bicheng Zhu [1,2,3,†], Paul Young [4,5], Yu Luo [6], John Taylor [4,5], Alan J. Cameron [2,3,4,5], Christopher J. Squire [4,5] and Jadranka Travas-Sejdic [1,2,3,*]

1 Centre for Innovative Materials and Health, School of Chemical Sciences, The University of Auckland, Private Bag 92019, Auckland 1142, New Zealand; rwan316@aucklanduni.ac.nz (R.W.); bicheng.zhu@auckland.ac.nz (B.Z.)
2 School of Chemical Sciences, The University of Auckland, Private Bag 92019, Auckland 1142, New Zealand; alan.cameron@auckland.ac.nz
3 MacDiarmid Institute for Advanced Materials and Nanotechnology, Victoria University of Wellington, Wellington 6012, New Zealand
4 School of Biological Sciences, The University of Auckland, Private Bag 92019, Auckland 1142, New Zealand; p.young@auckland.ac.nz (P.Y.); ja.taylor@auckland.ac.nz (J.T.); c.squire@auckland.ac.nz (C.J.S.)
5 Maurice Wilkins Centre for Molecular Biodiscovery, The University of Auckland, Auckland 1142, New Zealand
6 Micro- and Nano-Technology Research Center, State Key Laboratory for Manufacturing Systems Engineering, Xi'an Jiaotong University, Xi'an 710049, China; yuluo825@xjtu.edu.cn
* Correspondence: j.travas-sejdic@auckland.ac.nz
† These authors contributed equally to this work.

1. Chemicals and materials for EsterLigase-VHH E production pyrene-E-Tag synthesis

Fmoc-amino acids were purchased from CS Bio Ltd (Shanghai, China). Hydrazine hydrate and 1-[bis(dimethylamino)methylene]-1H-1,2,3-triazolo[4,5-b]pyridinium 3-oxide hexafluorophosphate (HATU) were purchased from (AK Scientific, Union City, CA, USA). N,N-dimethylformamide (DMF; AR grade) and acetonitrile (MeCN, HPLC grade) were purchased from Thermo Scientific (Hampshire, NH, USA). Diethyl ether (Et2O) was purchased from Avantor Performance Materials (Center Valley, USA) and dichloromethane were purchased from EP Ltd. (Auckland, New Zealand). Analytical reverse phase high-performance liquid chromatography (RP-HPLC) was performed on a Waters (Waltham, MA, USA) Alliance analytical HPLC equipped with a Phenomenex (Torrance, CA, USA) Luna C18 column (100 A, 5 μm, 4.6 mm x 250 mm) operated at room temperature, with chromatograms recorded at 214 nm and 254 nm. Semi-preparative RP-HPLC was performed on a Waters 1525 Binary HPLC pump equipped

with a Waters 2489 UV/visible detector (214 nm) using a Phenomenex Luna C18 semi-prep column (100 Å, 5 µm, 250 mm x 10 mm). For both analytical and semi-preparative RP-HPLC, solvents used were as follows: solvent A = 0.1% TFA in water (MQ H2O) and solvent B = 0.1% TFA in MeCN.

2. The calculation of the limit of detection

The calculation of the limit of detection (LOD) is based on the Signal-to-Noise Approach [1], where three times the sensor response from a blank buffer solution (PBS) was utilised. After measurements of the blank signal trice, the average value of $\Delta R_{ct}/R_{ct}^{0}$ was 0.0151 for LSGE, and the calibration equation for the LSG-based sensor was $\Delta R_{ct}/R_{ct}^{0} = 0.0866 \times \lg[\text{SP-RBD}]+1.0078$. Therefore, $\text{LoD}=10^{(3\text{x}\Delta R\text{ct}/R\text{ct0}-1.0078)/(0.0866)}=10^{(3\text{x}0.0151+1.0078)/(0.0866)}=7.68$ pM. The calculated LOD of the developed sensor was 7.68 pM.

A)

<u>MSYYHHHHHHDYDIPTTENLYFQ</u>GASQVQLVETGGGFVQPGGSLRLSCAASGVTLDYYAIGWFRQAP

GKEREGVSCIGSSDGRTYYSDSVKGRFTISRDNAKNTVYLQMNSLKPEDTAVYYCALTVGTYYSG

NYHYTCSDDMDYWGKGTQVTVSSGAGSGSGSGRVTNKKIVSSLQTTVEADGQSSTAEKSAEVTEN

KDGVNVVDTIHYKGLIPKQKYEVVGILYEVKDGKLVDPNKPITISNGTGEYTVSDSGEGEWKLNF

GKIDGVEARKSYVVYEEVTSVENLVDTDNDGNTS

B)

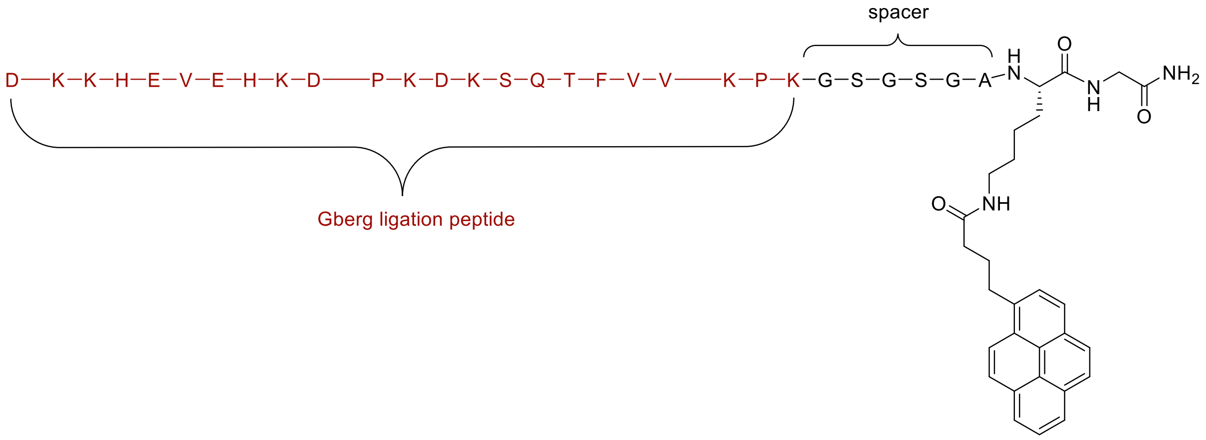

Figure S1. (**A**) Sequence of VHH E nanobody fused with EsterLigase. The N-terminal His[6] IMAC tag and rTEV protease recognition sequence are underlined and removed from the purified VHH E EsterLigase recombinant protein. The VHH E nanobody sequence (grey) is linked to the *Gemella bergeriae* (Gberg) EsterLigase domain (yellow) via a flexible linker (red). (**B**) Structure of pyrene-modified E-Tag. The E-Tag is composed of a Gberg ligation peptide with a C-terminal spacer that is conjugated to pyrene via the lysine N$^{\varepsilon}$ amino group.

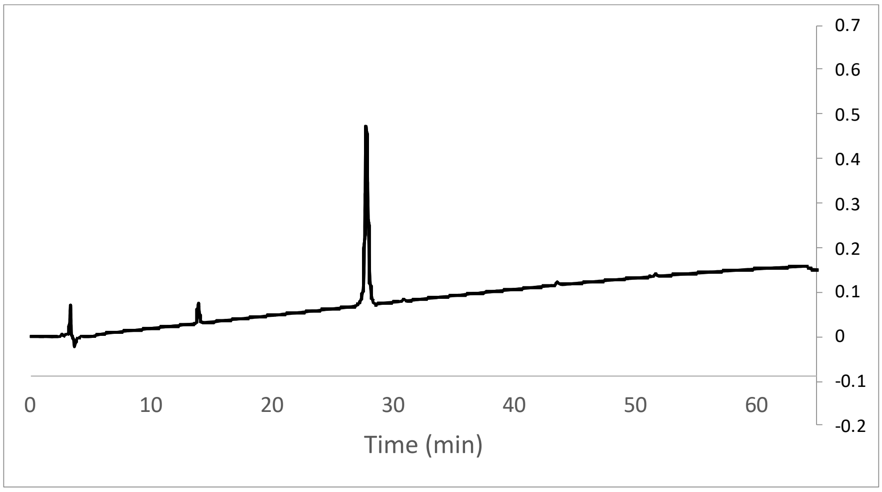

Figure S2. Analytical RP-HPLC for pyrene-modified E-Tag. RP-HPLC profile (214 nm) of pyrene modified E-Tag peptide. t_R 27.8 min, Phenomenex Luna C18 (100Å, 5 μm, 4.6 mm x 250 mm), linear gradient 5%B to 65%B (*ca.* 1%B/min) at 1 mL/min.

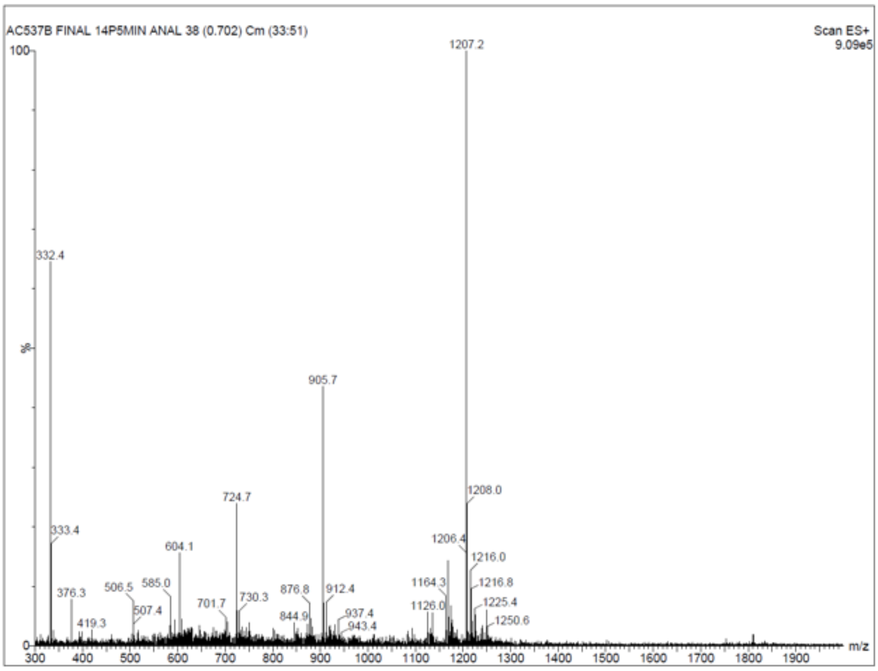

Figure S3. ESI-MS data for pyrene-modified E-Tag. Pyrene modified synthetic peptide ESI-MS (+ve) data. *m/z* calculated for [$C_{165}H_{253}N_{45}O_{47}$] 3617.9; mass observed deconv: 3618.6 ± 0.13. Charge states; 604.1 $[M+6H]^{6+}$, 724.7 $[M+5H]^{5+}$, 905.7 $[M+4H]^{4+}$, 1207.2 $[M+3H]^{3+}$.

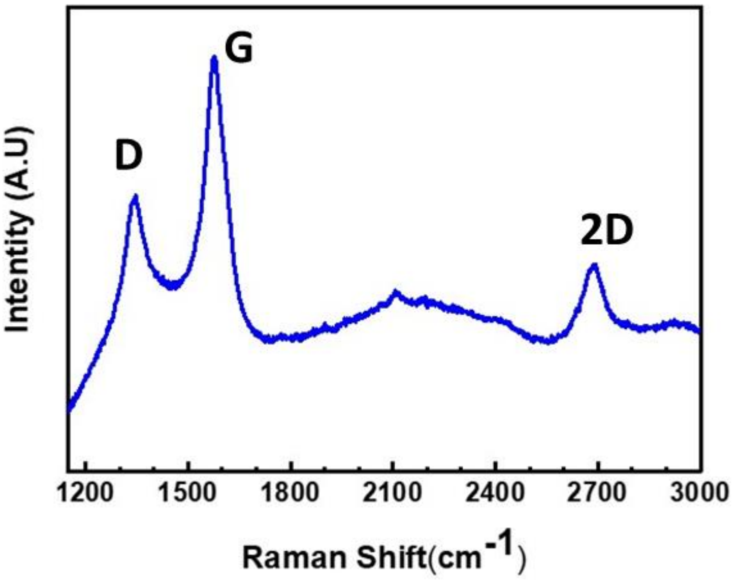

Figure S4. The Raman spectrum of the LSG. Excitation wavelength: 523 nm.

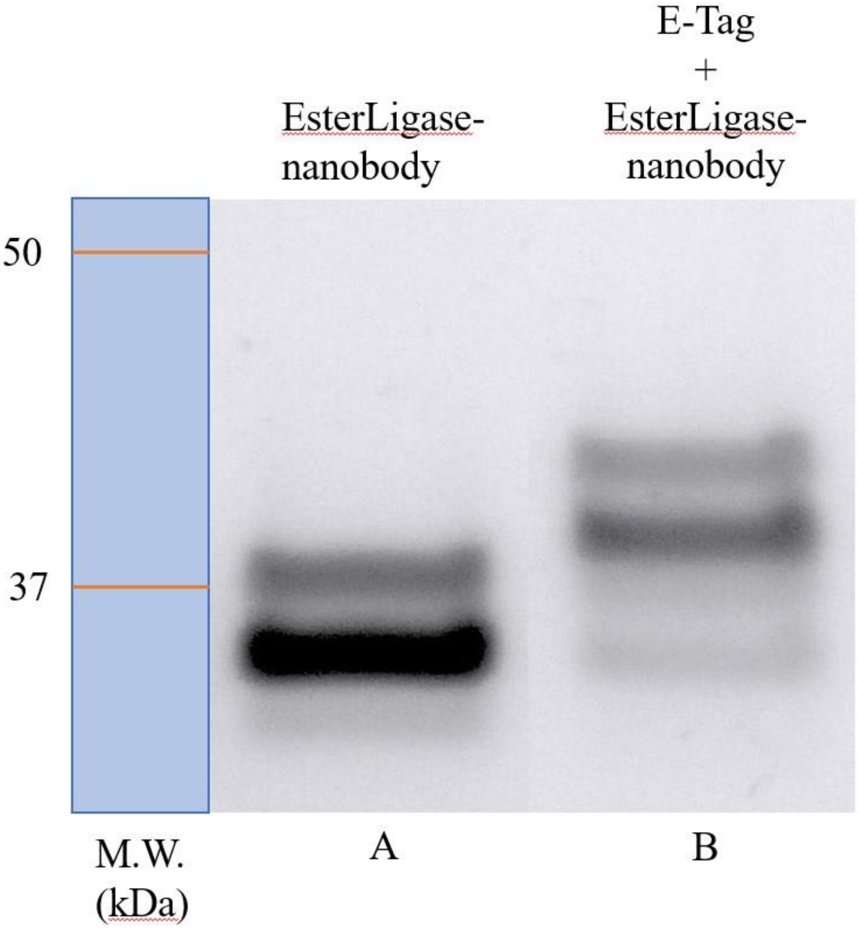

Figure S5. SDS-PAGE analysis (12% acrylamide gel) for ester bond formation. **Lane A**: EsterLigase-nanobody. **Lane B**: E-Tag and EsterLigase-nanobody allowed for overnight ligation reaction. Two species are visible from SDS-PAGE as the nanobody

is not fully reduced.

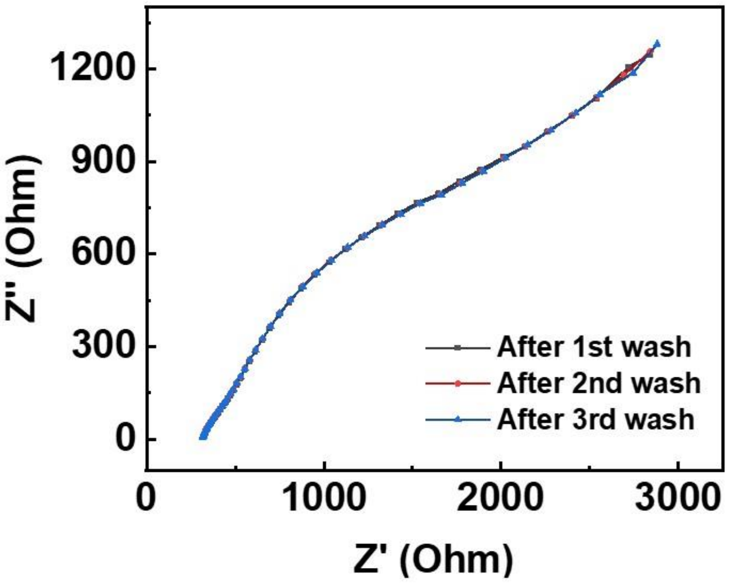

Figure S6. EIS spectra of LSG/PBA-pyrene-E-Tag linker/EsterLigase-nanobody after 1, 2 and 3 times washes with PBS buffer.

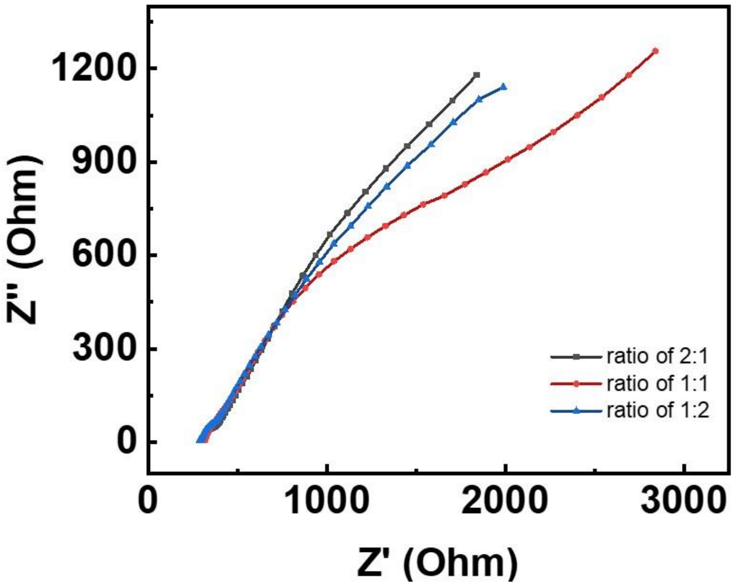

Figure S7. EIS spectra from the sensors with three different molar ratios of PBA to pyrene-E-Tag linker.

Reference

1. Armbruster, D.A.; Pry, T. Limit of blank, limit of detection and limit of quantitation. *Clin. Biochem. Rev.* **2008**, *29*, S49–S52.
